# Concerning Synthesis of New Biobased Polycarbonates with Curcumin in Replacement of Bisphenol A and Recycled Diphenyl Carbonate as Example of Circular Economy

**DOI:** 10.3390/polym13030361

**Published:** 2021-01-23

**Authors:** Vincenzo De Leo, Michele Casiello, Giuseppe Deluca, Pietro Cotugno, Lucia Catucci, Angelo Nacci, Caterina Fusco, Lucia D’Accolti

**Affiliations:** 1 Department of Chemistry, University of Bari, Via Orabona 4, 70126 Bari, Italy; michele.casiello@uniba.it (M.C.); giused56@gmail.com (G.D.); lucia.catucci@uniba.it (L.C.); angelo.nacci@uniba.it (A.N.); 2 Department of Biology, University of Bari, Via Orabona 4, 70126 Bari, Italy; pietro.cotugno@uniba.it; 3 ICCOM-CNR, Istituto di Chimica dei Composti Organometallici, Bari Section, Via Orabona 4, 70125 Bari, Italy

**Keywords:** curcumin, tetrahydrocurcumin, polycarbonate, trans polymerization, recycled diphenyl carbonate, recycled bisphenol A, biopolymers, UV barrier

## Abstract

Curcumin (CM) is a natural polyphenol well-known for its antioxidant and pharmaceutical properties, that can represent a renewable alternative to bisphenol A (BPA) for the synthesis of bio-based polycarbonates (PC). In the presented strategy, preparation of the CM-based PC was coupled with chemical recycling of the fossil-based BPA polycarbonate (BPA-PC) conducting a two-steps trans-polymerization that replaces BPA monomer with CM or its tetrahydrogenated colorless product (THCM). In the first step of synthetic strategy, depolymerization of commercial BPA-PC was carried out with phenol as nucleophile, according to our previous procedure based on zinc derivatives and ionic liquids as catalysts, thus producing quantitatively diphenyl carbonate (DPC) e BPA. In the second step, DPC underwent a melt transesterification with CM or THCM monomers affording the corresponding bio-based polycarbonates, CM-PC and THCM-PC, respectively. THCM was prepared by reducing natural bis-phenol with cyclohexene as a hydrogen donor and characterized by ^1^H-NMR and MS techniques. Polymerization reactions were monitored by infrared spectroscopy and average molecular weights and dispersity of the two biobased polymers THCM-PC and CM-PC were determined by means of gel permeation chromatography (GPC). Optical properties of the prepared polymers were also measured.

## 1. Introduction

The current plastics production system, almost completely based on fossil-based feedstock, poses significant economic, social, and environmental challenges. The omnipresent plastic pollution is a symptom of an inherently wasteful linear plastic economy, costing more than US$ 2.2 trillion per year in environmental and social damage [1,2,3]. To date, nearly 60% of this plastic waste is dumped into landfill and the environment, meanwhile at least 10% entering the oceans where plastics undergo phenomena of fragmentation and degradation posing serious risks to human health and the environment [3]. According to an European Commission report, the estimated annual release of microplastics into EU habitats alone, amounts to 75,000 to 300,000 tons [4].

The current model of the linear economy of plastic needs an urgent change of direction towards a more sustainable model of circular economy, under the pressure of an increased environmental awareness of citizens/consumers and policy makers, within a regulatory framework in which the productive and industrial world can move. The full implementation of the circular economy model hoped for plastics, benefits from the numerous possibilities offered by research in the field of polymers. The main approaches aim at (1) the production of plastic with renewable energy and feedstock, and (2) the design of consumer items to be used, reused, repaired and (mechanically, chemically or biologically) recycled in line with the waste hierarchy principles [4].

Plastic wastes can be considered a valuable resource that can be recycled by means of retro-polymerization to monomers obtaining two major advantages: the possibility of conserving large amounts of manufacturing energy which are trapped in the in polymer molecules and the possibility of obtaining virgin grade plastics at the end of the recycling process [5]. Alcoholysis, aminolysis, and hydrolysis are common procedures for depolymerization, typically performed under drastic conditions such as high pressure and temperatures, as well as high concentration of acids or bases during long time processing [6,7]. Polycarbonate (PC) is a thermoplastic polymer produced by the condensation reaction between bisphenol A (BPA) and a carbonyl source, generally phosgene or diphenyl carbonate (DPC) [8]. It has been used in several applications, for baby bottles and infant formula packages. However BPA is considered an endocrine disruptor and several studies have proposed a relationship between exposure to BPA and the occurrence of adverse health effects, such as cancer, infertility, diabetes and obesity [9]. For this reason, since 2011 BPA has been interdict in EU for the production of baby bottles, and restrictions are imposed when used in materials that come into contact with food [10]. Conversely, PC is largely used in construction for its mechanical resistance properties [11].

Several studies have been carried out for the chemical recycling of PC in order to reduce their environmental impact. In this regard, some of us developed an efficient and recyclable bifunctional catalyst composed by tetrabutylammonium chloride (TBACl) and zinc oxide nanoparticles (ZnO-NPs), useful for retro-polymerization of BPA polycarbonate (BPA-PC) in the presence of nucleophiles (including water, alcohols, and amines), leading to the complete recovery of BPA monomer and enabling the PC polymer to function as a green carbonylating agent (green phosgene alternative) for preparing carbonates, urethanes, and ureas [12]. This approach, however, is forced to the use of BPA as a recycled monomer because, to the best of our knowledge, no substitute for BPA has yet been found.

**Figure 1:**
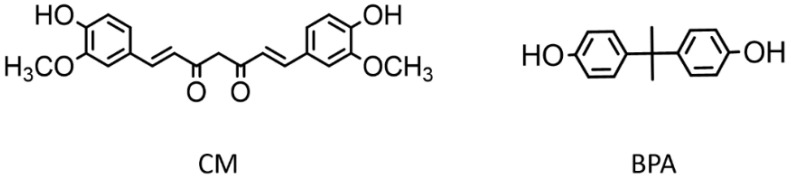
Structures of curcumin (CM) and bisphenol A (BPA).

To solve this problem, we decided to use a natural substance such as curcumin (CM) as a precursor for the synthesis of PCs, as its molecule shows structural analogies with BPA (Figure 1). CM or (1E,6E)-1,7-bis (4-hydroxy-3-methoxyphenyl)-1,6-heptadiene-3,5-dione is a yellow polyphenol naturally abundant in Turmeric plant (*Curcuma longa* L.) [13]. Numerous beneficial properties are recognized to this molecule, since many studies have confirmed its antioxidant, anti-inflammatory, antibacterial, and even anti-cancer properties. In order to increase CM water solubility, thus improving its stability and bioavailability, several strategies have been adopted, the main one being the inclusion in liposomes, micelles, solid lipid nanoparticles and in general in polymolecular aggregates of lipids, surfactants, and biopolymers, often in combination with each other [14,15,16].

In addition, because of its properties, CM has often been incorporated, functionalized, and conjugated to polymers as a dye, to improve the usability of CM in the field of drug delivery, or to obtain antibacterial, anticancer, and anti-inflammatory biocompatible materials [17,18,19,20]. Additionally, CM was used as a photoinitiator for the free radical photopolymerization of styrene and methacrylates [21,22]. On the contrary, only in a few cases CM has been used as a monomer for producing polymers, for example in the synthesis of polyesters by reaction with acid chlorides [13], in the polycondensation with diorganodichlorosilanes leading to poly[(arylenedioxy)(diorganylsilylene)]s [23], and in the synthesis of polyurethanes, where acted as a chain-extender [24]. The symmetric structure of CM molecule, indeed, reveals a natural vocation as an inherent monomer which can be readily polymerized by conventional methods, due to the two phenolic groups which are easily accessible, at the ends of molecule, and more nucleophilic than in BPA due to the presence of the electron-donor methoxy groups in the phenyl rings (Figure 1) [13].

**Figure 2:**
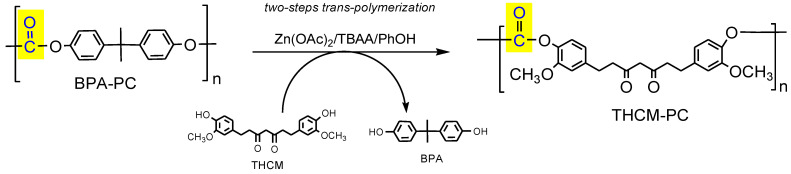
Scheme of the preparation process of THCM-PC, where the plastic waste BPA polycarbonate (BPA-PC) is converted into a renewable analogous in line with the needs of the circular economy. For the preparation of CM-PC, CM is used instead of tetrahydrogenated colorless product (THCM) in the trans-polymerization.

We report herein the synthesis of a new bio-based polycarbonate bearing CM or tetrahydro-curcumin (THCM) monomer in place of the toxic analogous BPA, by means of a two-steps trans-polymerization promoted by a bifunctional catalyst (zinc acetate/tetrabutylammonium acetate) under the assistance of phenol as nucleophile (Figure 2). According to a strategy that well embody the aims of the circular economy, this methodology not only transforms a toxic and fossil-derived waste plastic (BPA-PC) into a clean and renewable analogous polymer (THCM-PC or CM-PC), but also enables the carbonyl moiety to be recycled by transfer across the two polymers (operated by phenol as carrier), thus transforming a waste plastic into a safe carbonylating agent alternative to phosgene. 

## 2. Materials and Methods 

Curcumin (for synthesis, ≥75.0%), Cyclohexene, Pd/Carbon, Phenol, Tetrabutylammonium acetate, Zinc Acetate, all solvents were purchased from Sigma-Aldrich (St. Louis, MO, USA) and used as received, without any further treatment.

High Performance Thin Layer Chromatography (HPTLC) was performed on silica gel plates 10 × 10 cm, from Merck (Darmstadt, Germany). The plates were developed with chloroform: methanol (97:3 *v*/*v*). After scraping the silica in a band from the plate, the compound of interest was extracted rinsing the silica three times with chloroform: methanol (1:1, *v*/*v*). After centrifugation, the supernatants were combined and dried under a stream of N_2_ before subsequent analyses.

UV-Vis spectra of CM and its hydrogenated products were collected by a Varian (Palo Alto, CA, USA) Cary 5000 spectrophotometer in the range 200–500 nm.

UV-Vis-NIR spectra of prepared PCs were acquired casting the polymers on a quartz slide and collecting the measures by a Varian Cary 5000 spectrophotometer in the range 200–800 nm after letting to evaporate the solvent.

GC/MS analyses were run on a Shimadzu (Tokyo, Japan) GLC 17-A instrument connected with a Shimadzu QP5050A selective mass detector using a SLB-5MS column (30 m × 0.25 mm I.D., film thickness 0.25 nm). Mass spectra were performed in EI mode (70 eV) and conversions were determined using 2,5-dimethylanisole as an external standard. Identification of glycerol oligomers and determination of selectivities were performed by HPLC with a C18 Column Supelcosil (15 cm × 4.6 mm I.D., purchased from Sigma-Aldrich), a solvent program starting with H_2_O to Methanol for 40 min, and a flow rate of 1.0 mL/min, IT-TOF detector.

High-resolution mass spectra (HRMS) were obtained using a Shimadzu LCMS-IT-TOF instrument with the following settings: mass range 50–1000 m/z, ionization system electrospray ion source in positive ion mode, nebulizer gas nitrogen at 1.5 L/min, dry gas nitrogen at 102 MPa and 250 °C, collision gas argon.

ATR-FTIR spectra were recorded in the range of 400–4000 cm^−1^ on a Perkin Elmer (Waltham, MA, USA) spectrometer instrument.

NMR spectra were recorded on a Bruker (Billerica, MA, USA) 500 MHz spectrometer; the ^1^H-NMR (500 MHz) spectra were referenced to residual isotopic impurity of CDCl_3_ (7.25 ppm) and ^13^C-NMr (125 MHz) spectra were referenced to 77.0 ppm.

Gel permeation chromatography (GPC) was performed on an KNAUER S 2520 HPLC system equipped with TOSOH Corporation (Tokyo, Japan), columns (TSKgel G2000HHR, column size 7.8 mm I.D. × 30 cm, particle size 5 µm) and ultraviolet detector (wavelength: 254 nm). Tetrahydrofuran was used as the mobile phase at a flow rate of 1.0 mL min^−1^. Polystyrene standards were used for establishing the calibration curve. The PC polymers (~10 mg) were dissolved in tetrahydrofuran (10 mL) in duplicate. Bath sonication was employed to assist the dispersion and solution of each polymer, and the solutions were filtered through a 0.45 μm membrane filter before GPC analysis.

### 2.1. Depolymerization of PC Waste

BPA and DPC were obtained from PC waste modifying slightly a previously reported procedure [12,25]. Briefly, 1 mmol of polycarbonate corresponding to 0.254 g of polymer, calculated on the molecular weight of repeating unity (BPA-Carbonyl fragment, 254 Dalton) was dissolved in 10 mL of THF. To reaction mixture 2.63 g of phenol (28 mmol), 10 mg of Zn(OAc)_2_ (0.05 mmol, 5% respect to polycarbonate), and 0.301 g of tetrabutylammonium acetate were added. The reaction was carried out in a steel reactor for 3 h at 140 °C under magnetic stirring. The reacted mixture was filtered to remove (ZnOAc)_2_ co-catalyst (prompt to be re-used) and mixed with aqueous NaHCO_3_ under vigorous stirring. Then, after extraction with chloroform, the organic phase was dried over anhydrous Na_2_SO_4_, and evaporated to dryness. Recrystallization of the residue from chloroform and hexane gave DPC as a white solid in 98% of yield. ^1^H-NMR (400 MHz, CDCl_3_) δ 7.28–7.31 (m, 3H), 7.41–7.43 (m, 2H); ^13^C-NMR (400 MHz, CDCl_3_) δ 121.2, 126.58, 129.86, 151.28. MS m/z (%) 214 (M^+^, 75), 170 (50), 141 (55) 79 (100) [26]. In the basic solution remained BPA and residue of phenol salts dissolved, that were treated with a solution of HCl 5%. The mixture was extracted with Et_2_O and after evaporation the solid mixture was separated with column over silica gel with hexane/AcEt (80/20) as eluent. We obtained pure BPA as a white solid: ^1^H-NMR (400 MHz, CDCl_3_): δ 1.75 (s, 6H), 7.13–7.15 (d, 4H), 7.22–7.24 (d, 4H); ^13^C-NMR (400 MHz, CDCl_3_), δ 31, 43, 120, 128, 148, 152; GC–MS m/z (%): 228 (M^+^, 27), 213 (100), 119 (64), 43 (26), 99 (10), 91 (12), 65 (6). The depolymerization yield was calculated as previously described [12].

### 2.2. Synthesis of THCM

THCM was prepared according to Wagner and coworkers [27]. Briefly, in a 100 mL round bottom flask charged with methanol (21 mL), cyclohexene (3.6 mL), and 10% Pd/C (0.6 g), 0.3 g of commercial CM was added. A water reflux condenser was attached to the round bottom flask, and the mixture was heated to reflux at 80–82 °C under magnetic stirring for 6 h. The course of the reaction was monitored by UV-Vis spectroscopy. After that, the reaction solution was let cool to room temperature and filtered. The filtered solution was evaporated to give crude THCM by employing a rotary evaporator. The hydrogenation yield was 60%. ^1^H-NMR (CDCl_3_): δ 6.81 (d, 2H), 6.68 (s, 2H), 6.63 (d, 2H), 6.02 (s, 2H), 5.43 (s, 1H), 3.81 (s, 6H), 2.82 (m, 5H), 2.55 (m, 3H); IR spectrum (neat) 3600–3100, 2975, 2844, 1720, 1700, 1610, 1510, 1448, 1430, 1282, 1032. A part of the reaction mixture was purified by HPTLC in order to determine HRMS of THCM. After separation the HRMS [28] was determined: [M^+^Na^+^] C_21_H_24_O_6_ exp. 395.1465, calculated 395.1465.

### 2.3. Preparation of PC Polymers

PC polymers were prepared adapting a literature procedure [26]. 1.06 mmol of DPC and 1.0 mmol of relevant monomer, i.e., commercial CM (for CM-PC), THCM (for THCM-PC) or recovery BPA (for BPA-PC) were added at solid NaOH (2% in mol) as catalyst, using a three-necked flask, connected to a NaOH trap. The reactor was evacuated and purged with nitrogen, then the reaction mixture was heated at 150 °C for 3 h under nitrogen atmosphere. After this time, the reaction was gently cool down using a flow of N_2_, also useful to eliminate the phenol residue. Finally, the solid was dissolved in THF and crystallized by double distilled water. Polymerization yield was calculated by weighing the phenol released during the transpolymerization reaction as previously described [26]. Briefly, phenol was trapped in a 2N NaOH solution as it was removed from the nitrogen stream. This solution was subsequently acidified with 2N HCl up to pH = 3.0–4.0. The solution was subsequently subjected to extraction with AcEt and evaporation under vacuum at room temperature. The residual phenol was weighed to obtain W_Ph_. The calculation was done with the formula: Yield = W_Ph_/W_Calc_, where W_Calc_ is the theoretically obtainable weight of phenol. All the polymers were characterized using UV-Vis-NIR, IR and ^1^H-NMR spectroscopy. The medium molecular weight was determined using GPC. The polycarbonates were dissolved in tetrahydrofuran, and 10 mg of each PC samples in duplicate were dissolved in 10 mL THF. Bath sonication was employed to assist the dispersion and solution of each polymers. The solutions were filtered through a 0.45 μm membrane filter before GPC analysis. 

## 3. Results and Discussion

### 3.1. Synthesis of THCM

**Figure 3:**
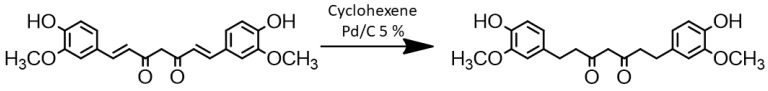
CM reduction process to obtain THCM.

Due to its chemical structure, CM appeared to us to be a useful synthetic equivalent of BPA, although its intense yellow colour did not recommend its use as monomer for PC synthesis. To circumvent this problem, the hydrogenated derivative THCM was prepared by selective reduction of the two double bonds with a known procedure [27] employing cyclohexene as a cheap and sacrificial reducing agent and Pd/C catalyst (Figure 3).

**Figure 4:**
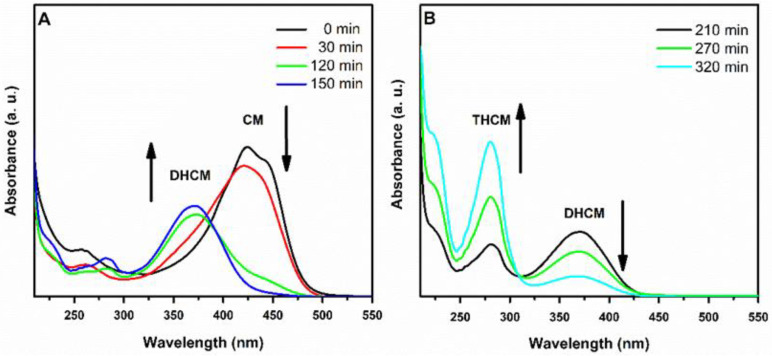
Monitoring of the CM reduction process over time (0–150 min, panel (**A**); 210–320 min, panel (**B**)) by UV-Vis spectroscopy.

Reduction reaction was monitored over time by UV-Vis spectroscopy (Figure 4). During the reaction progress, the characteristic peak of CM at 420 nm dropped in favor of that of dihydrocurcumin (DHCM) at 373 nm [29]. After about three h, the 280 nm signal characteristic of THCM appeared. Reaction was stopped immediately at the disappearance of the DHCM peak to avoid further unwanted reduction processes. THCM was then purified by means of preparative HPTLC for subsequent characterizations.

**Figure 5:**
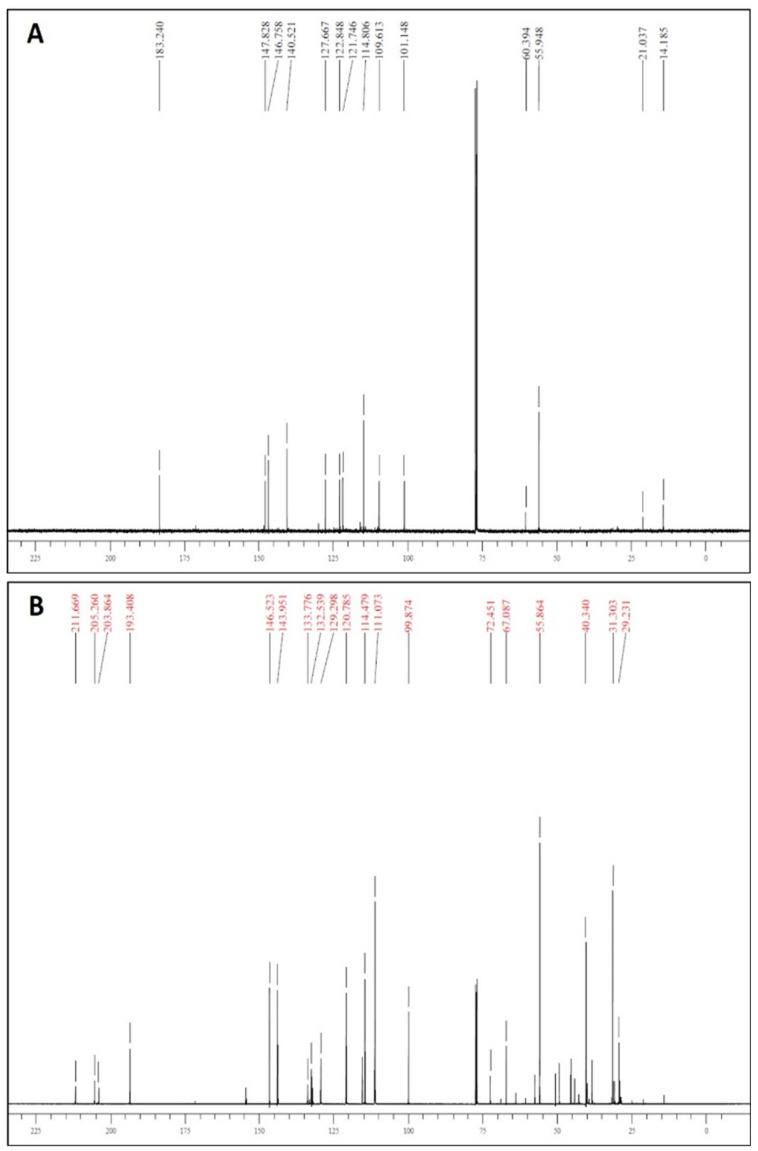
^13^C-NMR spectra of CM (panel **A**) and THCM (panel **B**).

^13^C-NMR spectra confirmed the presence of unconjugated carbonyls in the 203–206 ppm range, and the appearance of diagnostic signals of THCM in the 29.23–40.34 ppm range (Figure 5). Additionally, HR-MS spectra confirmed the identity of the tetrahydrogenated product (see Section 2.2).

The conversion of CM was >90% and after the workup operations THCM was used as it is for the synthesis of the polymer (THCM-PC).

### 3.2. Preparation of Polycarbonates

**Figure 6:**
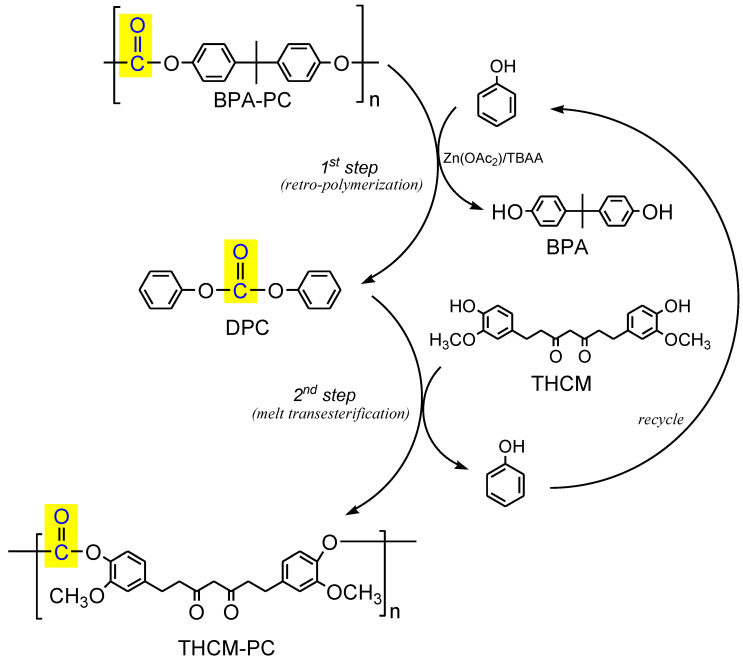
Scheme of trans-polymerization process for the synthesis of curcumin-based polycarbonates. For the preparation of CM-PC, CM is used instead of THCM in the trans-esterification.

CM and THCM were both used for a two-steps preparation of PCs, respectively CM-PC and THCM-PC, performing a retro-polymerization of BPA-PC with phenol followed by melt transesterification of DPC intermediate with THCM or CM (see procedure reported in Section 2.3). As a whole, the process can be considered a trans-polymerization that transforms the plastic waste BPA-PC into bio-based analogous polymers, replacing the toxic BPA with renewable and clean monomers (Figure 6). In the first step, BPA-PC is catalytically depolymerized with phenol as nucleophile to produce quantitatively DPC and BPA. After purification, DPC reacts with THCM or CM affording THCM-PC or CM-PC, releasing phenol. The latter in turn can be recovered and virtually reused for a new run. Furthermore, this procedure also allows the recovery of BPA in high purity, thus closing the cycle of a complete chemical recycling of the widely produced plastic waste BPA-PC.

The depolymerization reaction was carried out by modifying the previous protocol based on ZnO nanoparticles and tetrabutylammonium chloride [12]. In this case, to make the procedure scalable at an industrial level, more accessible products such as bulk Zn(OAc)_2_ and tetrabutylammonium acetate (TBAA) were chosen as substitutes of the former nanostructured binary catalysts. Zn(OAc)_2_, being insoluble in THF, can be recovered by filtration of the reaction mixture at the end of depolymerization. After washing with fresh THF and drying under a nitrogen stream, the recovered Zn(OAc)_2_ can be promptly reused by adding fresh reagents and TBAA co-catalyst.

**Figure 7:**
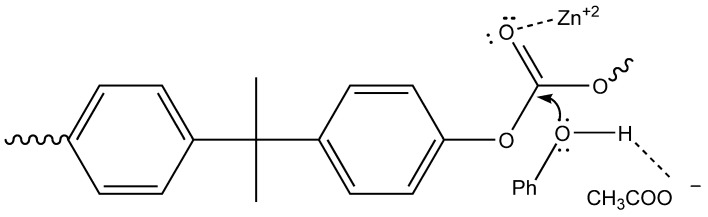
Catalytic depolymerization process of polycarbonate (PC) waste for the recovery of diphenyl carbonate (DPC).

Efficiency of such a system was ascribed to the synergic combination of Zn^2+^, that enhanced the electrophilicity of carbonyl moiety by oxygen coordination, and tetrabutylammonium acetate, which increased nucleophilicity of phenol due to the H-bond accepting properties of anion, thus enabling depolymerization at milder temperature conditions (Figure 7). In this way, a very high depolymerization yield (about 96%) can be obtained.

**Figure 8:**
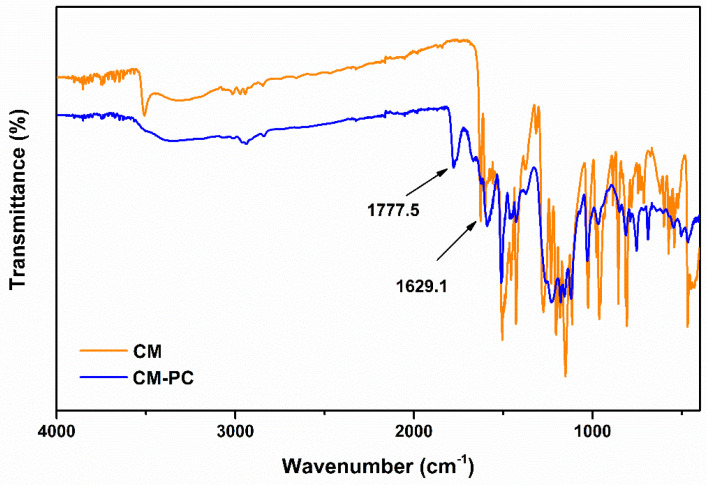
IR spectra of CM and CM-PC polymer.

**Figure 9:**
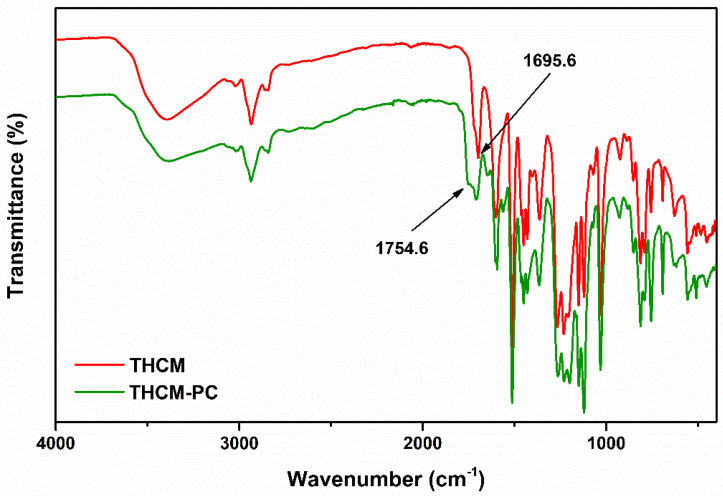
IR spectra of THCM and THCM-PC polymer.

Analysis of the IR spectra of reaction mixture (Figure 8 and Figure 9) highlights the formation of carbonate compounds, thus confirming the successful outcome of the polymerization. These signals included in the 1770–1780 range and attributable to the carbonyl functionality of the carbonate [30] are clearly identifiable both in the CM-PC (Figure 8 and Table 1) and in the THCM-PC (Figure 9 and Table 2) spectra. Furthermore, the C=O stretching signals of the ketone functions are also present. In the case of CM and CM-PC this signal fell to 1629 cm^−1^ (Figure 8) due to the conjugation with the double bond, whereas in the case of THCM and THCM-PC this value rose to almost 1700 cm^−1^ (Figure 9).

Table 3 shows the polymerization yield, the polystyrene-equivalent average molecular weights and the dispersity values of THCM-PC and CM-PC measured by means of GPC. For comparison, the values related to BPA-PC polymerized starting from BPA and DPC are also reported, both coming from the depolymerization of PC waste. Polymerization yields were found to be high for both PCs derived from CM and comparable to that of BPA-PC. The molecular weight data obtained show that both polymers prepared from CM have a molecular weight higher than that of BPA-PC, in particular THCM-PC almost twice. All the PC polymers prepared have a certain degree of dispersity, as they showed Ð values > 1, moreover the THCM-PC showed the highest dispersity value, in accordance with the broader molecular weight [31,32].

**Figure 10:**
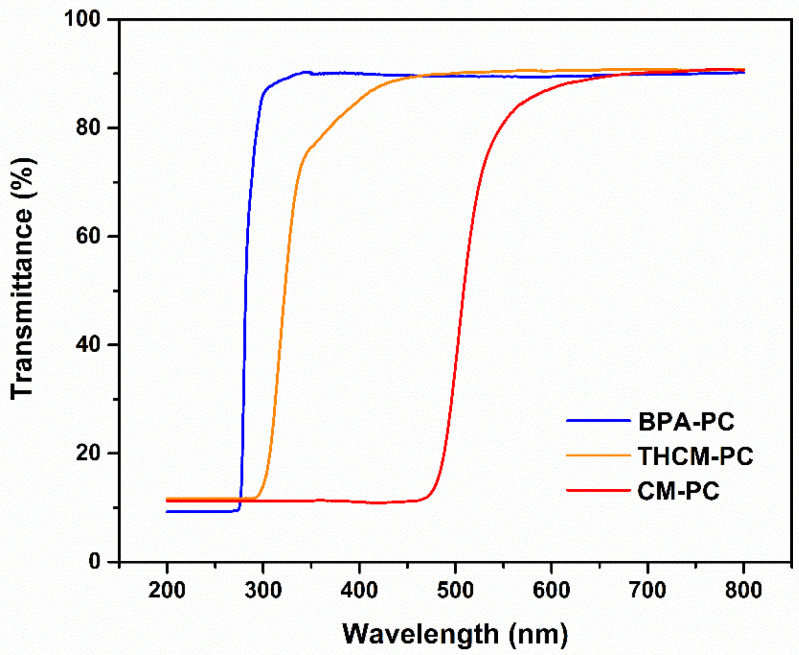
Transmittance spectra of BPA-PC, CM-PC, and THCM-PC polymer.

Figure 10 shows the transmittance spectra of CM based PCs acquired in the UV-Vis-NIR region. For comparison, the spectrum of BPA-PC is also reported. The latter is at the same time representative of the commercial one. The spectrum of THCM-PC is very similar to that of BPA-PC even if the transmittance begins to decrease at slightly higher values of wavelength, but always in the ultraviolet region, since THCM has a maximum absorption at 280 nm. This characteristic could be useful for some applications to keep the screen effect higher in the UV region. For this purpose, indeed, CM was sometimes mixed as an additive to polymers [33]. Analysing the spectrum of the CM-PC in fact, it is possible to notice how the UV region is totally shielded as curcumin has an intense and broad absorption between 300 and 500 nm. However, CM-PC also maintains a good transparency as the transmittance at λ > 550 nm remains high, at the same values of BPA-PC.

The peculiar properties of CM could also positively influence other characteristics of the prepared PCs. Previous studies have indeed shown that the antioxidant and biocidal properties of CM can be used in the field of polymers by improving their characteristics. In particular, it has been shown that curcumin is a more efficient melt and thermo-oxidative stabilizer of polyethylene than the synthetic phenolic antioxidant used as reference [34] and that CM can be advantageously used in the synthesis of rigid polyurethane foams with enhanced mechanical, antibacterial, and anti-aging properties [35,36]. Similarly, we expect that the use of CM in the synthesis of PCs can bring advantages, especially in terms of antioxidant properties, even if further measures and more in-depth characterizations are obviously necessary.

## 4. Conclusions

In the present study we proposed for the first time the use of the natural molecule CM for the replacement of BPA in the synthesis of PC polymers. In this strategy, preparation of the CM-based PCs was coupled with chemical recycling, included the carbonyl moiety, of the fossil-based BPA-PC in a two-steps trans-polymerization process. In the first step, BPA-PC is catalytically depolymerized with phenol as nucleophile to produce quantitatively diphenyl carbonate DPC, that in turn gives rise, in the second step, a melt transesterification with CM or its tetrahydrogenated product THCM. 

The possibility of using CM or its derivatives as building blocks for polymer preparation, opens the way to the replacement of molecules of fossil origin and worrying from a toxicological and ecotoxicological point of view. Even if at present the industrial scalability of the proposed synthesis is complicated by the higher cost of CM, which is currently marketed as a food grade or research grade material, we are convinced that under the pressure of international legislation and the increased environmental sensitivity of consumers, natural alternatives such as CM may become accessible also to the polymer industry. Many legislations indeed, such as the REACH regulation in the European Union area, impose severe restrictions on the use of molecules that have proven to be carcinogenic and endocrine disruptors such as BPA, while promoting the search for safer and more sustainable alternatives. Under this impulse, increased production and exploitation of waste and non-edible parts of turmeric could lead to rapid lowering of costs for CM of industry grade in the near future. Furthermore, the coupling of bio-based polymer synthesis with the recycling of widely produced plastic wastes aligns with the demands of an increasingly urgent circular economy model. While further studies are certainly needed, this research may prove to be of great interest to the world of modern polymer entrepreneurship.

## Figures and Tables

**Table 1:** Attribution of signals relating to the IR spectra in the Figure 8.

Frequency (cm^−1^)	Attributions
3600–3100	O–H stretching
3000–3100	Aromatic C–H stretching
2975–2845	Symmetric and asymmetric aliphatic stretching
1777	C=O carbonate stretching
1629	C=O ketone stretching
1510	Bending C=C–H; aromatic bending C–C

**Table 2:** Attribution of signals relating to the IR spectra in the Figure 9.

Frequency (cm^−1^)	Attributions
3600–3100	O–H stretching
3000–3100	Aromatic C–H stretching
2975–2845	Symmetric and asymmetric aliphatic stretching
1754	C=O carbonate stretching
1695	C=O ketone stretching
1510	Bending C=C–H; aromatic bending C–C

**Table 3:** Polymerization yield of prepared PCs and related polystyrene-equivalent average molecular weights and dispersity values measured by gel permeation chromatography (GPC).

Polymer	Yield (%)	M_w_ (g mol^−1^)	M_n_ (g mol^−1^)	Ð
THCM-PC	82	1218	764	1.59
CM-PC	78	1019	673	1.51
BPA-PC	86	679	572	1.19

M_w_—mass-average molecular weight; M_n_—number-average molecular weight; Ð—dispersity.

## Data Availability

Not applicable.
